# Post-initiation chlorophyllin exposure does not modulate aflatoxin-induced foci in the liver and colon of rats

**DOI:** 10.1186/1477-3163-5-6

**Published:** 2006-02-06

**Authors:** Gayle A Orner, Bill D Roebuck, Roderick H Dashwood, George S Bailey

**Affiliations:** 1 Linus Pauling Institute, Oregon State University, Corvallis, OR, USA; 2 Department of Pharmacology and Toxicology, Dartmouth Medical School, Hanover, NH, USA; 3 Environmental and Molecular Toxicology Department, Oregon State University, Corvallis, OR, USA

## Abstract

Chlorophyllin (CHL) is a promising chemopreventive agent believed to block cancer primarily by inhibiting carcinogen uptake through the formation of molecular complexes with the carcinogens. However, recent studies suggest that CHL may have additional biological effects particularly when given after the period of carcinogen treatment. This study examines the post-initiation effects of CHL towards aflatoxin B1 (AFB_1_)-induced preneoplastic foci of the liver and colon. The single concentration of CHL tested in this study (0.1% in the drinking water) had no significant effects on AFB_1_-induced foci of the liver and colons of rats.

## Background

During the past decade chlorophyllin (CHL) has progressed from initial *in vitro *anti-mutagenicity experiments to *in vivo *studies of anti-tumor mechanisms in trout, mice, and rats [reviewed by [1]], and into successful chemoprevention trials in humans exposed to dietary aflatoxin B_1 _(AFB_1_) [2]. An important mechanism for the protective effects of CHL as a blocking agent appears to be the ability of this planar compound to complex with carcinogens, thus preventing carcinogen-DNA adduction. However, several recent studies focusing on *post*-initiation effects of CHL suggest that CHL may have additional effects independent of molecular complex formation. In one report, CHL caused a concentration dependent suppression of liver tumor formation in rats treated with the heterocyclic amine, 2-amino-3-methylimidazo [4,5-*f*]quinoline (IQ) [3]. In that study, CHL treatment was started 1 week after the last dose of carcinogen, suggesting that CHL might be effective as a suppressing agent in the liver. Although CHL suppressed IQ-induced liver carcinogenesis in the rat, deleterious effects have been reported in the colon, including tumor promotion in some studies [3,4]. These additional biological properties of CHL could possibly be mediated through effects on apoptosis [5] or drug metabolism [6-8].

In the present study, we examined the post-initiation effects of CHL towards AFB_1_-induced putative preneoplastic foci of the liver and colon.

## Methods

### Animals

Male F344 rats, 3 weeks of age were purchased from the National Cancer Institute (Frederick, MD) and housed two per cage in shoebox cages at 22° on a 12 hr light/dark cycle. All rats were fed AIN-93G diet (Dyets Inc., Bethleham, PA) *ad libitum*. Prior to and during the period of carcinogen administration, the diet was free of ethoxyquin since this antioxidant prevents the formation of putative preneoplastic foci in rat livers [9]. Cardboard tubes were provided for environmental enrichment. Rats were weighed weekly throughout the experiment.

### Chemicals

CHL was a gift from Dr. T.W. Kensler and was lot-matched to the CHL preparation used in the human clinical trial [2]. AFB_1 _was obtained from Aldrich (Milwaukee, WI). All other chemicals were obtained from Sigma-Aldrich (St. Louis, MO).

### Experimental design

**Figure 1:**
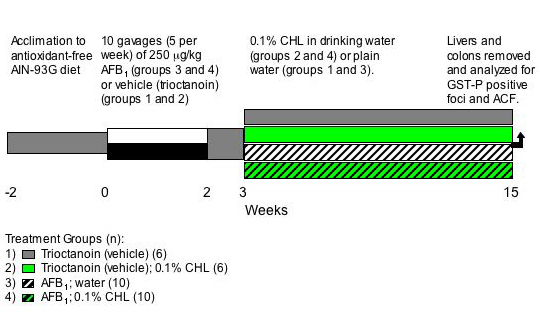
Experimental protocol for evaluation of the effects of post-initiation CHL on the development of hepatic GST-P positive foci and colonic ACF. Rats received 250 μg AFB_1 _per kg body weight five times a week for two weeks. Beginning one week after the end of the initiation period and continuing for 12 weeks, half of the animals were given 0.1% CHL in their drinking water.

Following a two-week acclimation to antioxidant free AIN-93G diet, rats were randomly assigned to one of 4 treatment groups as shown in figure 1. Rats were given trioctanoin (groups 1 and 2) or 250 μg/kg AFB_1 _in trioctanoin (groups 3 and 4) five times per week for two weeks by oral gavage. Beginning one week after the last AFB_1 _gavage, and continuing for 12 weeks, groups 2 and 4 were given 0.1% CHL in their drinking water. Fresh CHL solutions were prepared and administered every other day. After 12 weeks of CHL treatment rats were euthanized with carbon dioxide and livers and colons removed.

### Quantification of GST-P positive hepatic foci

Livers were sliced into multiple 3 mm sections, fixed in acetone, and processed for histology using the AmesX fixation and processing procedure [10]. Slides were stained for expression of GST-P and examined by light microscopy as previously described [11]. The volume percent of liver occupied by GST-P positive foci is considered the least biased and most analogous to tumor burden [12]; therefore, this was the primary endpoint evaluated.

### Quantification of colonic ACF

Colons were removed, washed with chilled phosphate buffered saline, fixed mucosa side up in 10% phosphate buffered formalin, stained with 0.2% methylene blue, and ACF scored as previously described [13]. All samples were coded so that the individual analyzing them was blinded to the treatment group and animal numbers.

### Statistical analysis

Results are expressed as means ± S.D. within a treatment group. Data were evaluated by analysis of variance (ANOVA) with post-hoc Tukey's and Bonferroni multiple comparison tests. Analyses were performed using the SAS or StatView statistical packages (SAS Institute, Cary, NC).

## Results

**Figure 2:**
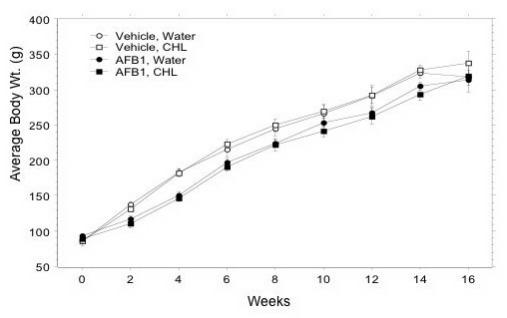
Effects of CHL and AFB_1 _on body weights. Data are mean (± SE) at each timepoint.

Rats treated with AFB_1 _had significantly lower growth rates than vehicle-treated animals; however, there were no significant differences in growth rates (Fig. 2) or food or liquid consumption (not shown) between the water and CHL-treated rats.

**Figure 3:**
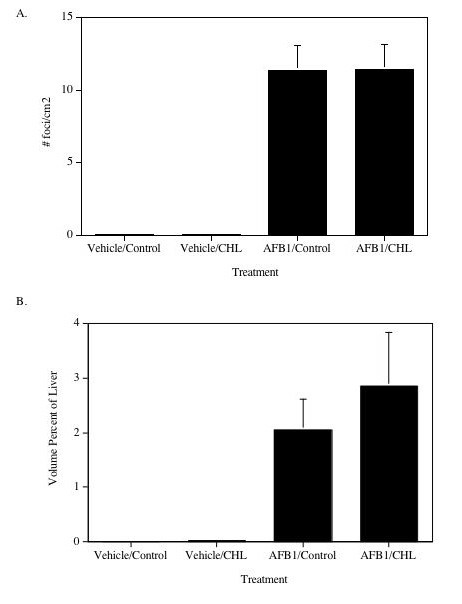
Effects of CHL and AFB_1 _on liver GST-P positive foci. Data are mean (± SE) for each treatment group. (A) No. Foci per cm^2^. (B) Volume percent of liver occupied by foci.

GST-P positive foci were seldom observed in vehicle-treated rats (0.07 foci/cm^2 ^liver examined); but were common in AFB_1_-treated rats (11 foci/cm^2^) (Fig. 3A). Twelve weeks of post-initiation treatment with 0.1% CHL had no significant effects on AFB_1_-induced GST-P positive focal density (Fig. 3A) or volume percent of liver occupied with GST-P positive foci (Fig. 3B).

**Figure 4:**
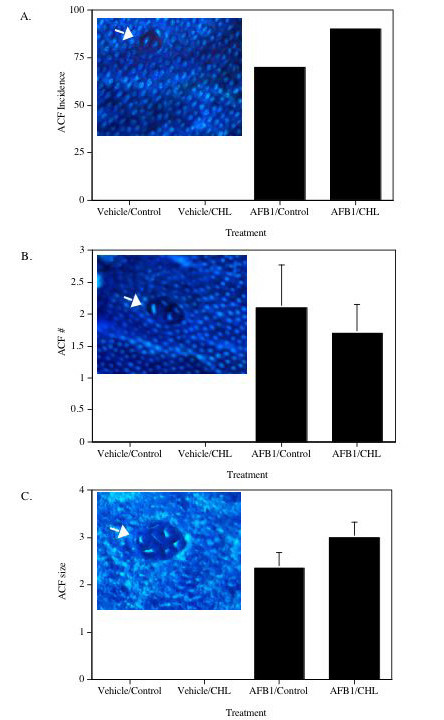
Effects of CHL and AFB_1 _on colonic ACF. Data are mean (± SE) for each treatment group. (A) Incidence (% of animals with ACF). (B) Number of ACF per colon. (C) Size (# crypts) of ACF. Insert photos show examples of foci containing (A) two, (B) four, and (C) seven aberrant crypts.

The AFB_1 _treatment regime utilized in this study resulted in aberrant crypt foci (ACF) in colons of 80% of AFB_1_-treated rats (Examples are shown in Figure 4). These putative preneoplastic foci were not found in the colons of vehicle-treated animals. Post-initiation treatment with 0.1% CHL had no significant effect on the incidence, multiplicity, or size of ACF (Figure 4A–C).

## Discussion/conclusion

CHL is a promising chemopreventive agent that is believed to block the bioavailability of certain carcinogens by forming complexes with the carcinogen. CHL is a highly effective blocking agent in animal models and was recently shown to be effective at reducing urinary aflatoxin-DNA adduct biomarkers in humans exposed to high levels of dietary aflatoxins [2]. In addition to the well-established carcinogen blocking mechanism of CHL, some studies suggest that CHL may have additional biological properties. The dose-dependent suppression of IQ-induced liver tumor formation by CHL [3] raises the exciting possibility that the benefit of CHL towards hepatocarcinogenesis could be even greater than predicted by reductions in DNA adduct formation. However, the mixed results of CHL towards colon carcinogenesis [3,4,14] suggest that under certain circumstances CHL may have detrimental effects.

Although AFB_1 _is primarily considered a liver carcinogen, lifetime exposure also produces colon tumors in rats, particularly in vitamin A deficient animals [15-17]. Based on two case reports of aggressive colon cancer in humans occupationally exposed to aflatoxins [18] and the presence of AFB_1 _DNA-adducts in colorectal cancer tissue from patients in the United Kingdom [19], the colon may also be a target organ of AFB_1 _carcinogenesis, particularly in parts of the world where hepatitis B infection is uncommon. Although the primary purpose of this study was to examine the post-initiation effects of CHL on preneoplastic foci of the liver, this experiment also provided an opportunity to determine if the AFB_1 _exposure protocol routinely used to produce altered hepatic foci in rats also results in preneoplastic lesions of the colon. Indeed, this AFB_1 _exposure produces a high incidence and multiplicity of ACF and provides a unique opportunity to simultaneously examine the effects of chemopreventive agents on preneoplastic foci of both the liver and colon.

Hepatic GST-P positive foci and colonic ACF are well-established markers for estimating the effect of chemopreventive agents on tumor outcome. GST-P positive foci accurately predicted the protective effects of oltipraz towards AFB_1_-induced hepatocarcinogenesis [20] and the potency of over 60 chemopreventive agents at inhibiting ACF correlates extremely well with their effects on tumor formation [21].

However, the process of tumor development is complex and our understanding of which preneoplastic markers will progress to tumors is incomplete. Recently the role of ACF as precancerous lesions has been challenged by studies suggesting that mucin depleted foci [22,23], beta-catenin accumulated crypts [24,25], or other atypical foci [26] may be of greater value at predicting tumor formation than the typical ACF first described by Bird in 1987 [27]. While the strong correlation between ACF and colorectal cancer in laboratory animals [21] and their presence in humans at risk for colorectal cancer [28-30] continue to support the utility of these foci for predicting tumor outcome, we recognize that no preneoplastic markers can fully replace tumors as the ultimate endpoint. Therefore, additional studies examining the post-initiation effects of CHL towards AFB_1_-initiated liver and colon cancer may be appropriate as the use of this chemopreventive agent becomes more widespread. However, the current study suggests that post-initiation treatment with 0.1% CHL is unlikely to modulate liver or colon tumor formation based on the lack of effects towards the formation of AFB_1_-induced hepatic GST-P positive foci and colonic ACF.

A major limitation of this study is that only a single concentration of CHL (0.1%) was tested. This concentration was selected because 0.1% CHL is the concentration that is most effective at inhibiting IQ induced liver tumors [3].

However, promotion of DMH-induced colon tumors occurs at much lower CHL concentrations (0.001%) [3]. Additional studies examining a range of CHL concentrations are necessary before concluding that CHL does not enhance AFB_1_-induced colon carcinogenesis. However, this study demonstrates that the protocol used to examine effects of chemopreventive agents towards AFB_1_-induced altered hepatic foci can include preneoplastic foci of the colon as an additional endpoint.

## Authors' contributions

GAO assisted in the design of the project, performed the animal study, performed the ACF scoring, and drafted the manuscript. BDR assisted in the design of the study, supervised the GST-P foci scoring and statistical analysis, and helped to draft the manuscript. RHD assisted in the design of the project, and helped to draft the manuscript. GSB conceived of the study, participated in its design, and helped to draft the manuscript.
